# Supplementary material for: Climate is stronger than you think: Exploring functional planting and TRIAD zoning for increased forest resilience to extreme disturbances
Source: PLoS One. 2025 Jun 27;20(6):e0326627. doi: 10.1371/journal.pone.0326627 (PMC12204526; doi:10.1371/journal.pone.0326627)
Supplement: S1 File — (DOCX) [file pone.0326627.s001.docx]

Climate is stronger than you think: Exploring functional planting and TRIAD zoning for increased forest resilience to extreme disturbances

Supplementary Material

Clément Hardy^1*^, Christian Messier^1^, Yan Boulanger^2^, Dominic Cyr^3^, Élise Filotas^4^

^1^Department of Biological Sciences, Université du Québec à Montréal, Canada

^2^Natural Resources Canada, Canadian Forest Service, Laurentian Forestry Centre, Québec, QC, Canada

^3^Sciences et Technology Branch, Environment and Climate Change Canada, Gatineau, QC, Canada

^4^Department of Science and Technology of the TELUQ, Université du Québec, Canada

*Corresponding author: clem.hardy@outlook.fr

Table of Contents

[A Species used and their life history traits 3](#__RefHeading___Toc11266_1416780302)

[B Details of the prescriptions implemented in Biomass Harvest 4](#__RefHeading___Toc3401_765435977)

[B.1 Control of each prescriptions spatial distribution through the python script 4](#__RefHeading___Toc5622_1855782233)

[B.2 Clear cuts (CC-PlantFunct, CC-PlantIntens, CC-NormalPlant and CC-NoPlant) 5](#__RefHeading___Toc5624_1855782233)

[B.2.1 CC-PlantFunct 5](#__RefHeading___Toc5626_1855782233)

[B.2.2 CC-PlantIntens 7](#__RefHeading___Toc5628_1855782233)

[B.2.3 CC-NormalPlant 8](#__RefHeading___Toc5630_1855782233)

[B.2.4 CC-NoPlant 8](#__RefHeading___Toc5632_1855782233)

[B.3 Selection cuts (SC) 9](#__RefHeading___Toc5634_1855782233)

[B.4 Commercial Thinning (CT) 10](#__RefHeading___Toc5636_1855782233)

[C Details of the catastrophic events – Distribution and effects 11](#__RefHeading___Toc11752_1540369786)

[C.1 Large Fire 11](#__RefHeading___Toc5638_1855782233)

[C.2 Severe Drought 13](#__RefHeading___Toc5640_1855782233)

[C.3 Mountain Pine Beetle epidemic 13](#__RefHeading___Toc5642_1855782233)

[D Clustering to create the functional groups 14](#__RefHeading___Toc5644_1855782233)

[E Surface burned in the different scenarios 17](#__RefHeading___Toc5646_1855782233)

[F Evaluating the size of the differences in the net change of the different management scenarios 21](#__RefHeading___Toc5648_1855782233)

[G Differences in the dynamic of the mature biomass in the north and in the south of our study area 23](#__RefHeading___Toc5650_1855782233)

## Species used and their life history traits

The species and their associated life-history traits that we used are the same as those in the study by Tremblay et al. (1) are detailed in Table 1, except for two hybrid species that we added in our simulation. These parameters were derived from various sources (e.g., 2, 3) and from expert judgment when empirical sources did not exist (1). The parameters of the two hybrid species (the hybrid poplar POPU.HYB and hybrid larch LARI.HYB) were copied from their non-hybrid counterparts (POPY.TRE and LARI.LAR respectively), but were given a smaller longevity and were incapable of dispersing by themselves. This was done based on expert opinion to imitate the fast-growing nature of both hybrids, and the fact that their dispersion would only be done through human planting and heavy fertilization.

| **Real name** | **Code name** | **Longevity (year)** | **Age of sexual maturity** | **Shade tolerance** | **Fire tolerance** | **Seed dispersal distance** | | **Vegetative reproduction probability** | **Sprout age** | | **Post fire regeneration** |
| --- | --- | --- | --- | --- | --- | --- | --- | --- | --- | --- | --- |
|  |  |  |  |  |  | **Effective** | **Max** |  | **Min** | **Max** |  |
| *Abies balsamea* | ABIE.BAL | 150 | 30 | 5 | 1 | 25 | 160 | 0 | 0 | 0 | none |
| *Acer rubrum* | ACER.RUB | 150 | 10 | 3 | 2 | 100 | 200 | 0.5 | 10 | 100 | resprout |
| *Acer saccharum* | ACER.SAH | 300 | 40 | 5 | 2 | 100 | 200 | 0.1 | 10 | 60 | resprout |
| *Betula alleghaniensis* | BETU.ALL | 300 | 40 | 3 | 1 | 100 | 400 | 0.1 | 10 | 180 | resprout |
| *Betula papyrifera* | BETU.PAP | 150 | 20 | 2 | 1 | 200 | 5000 | 0.5 | 10 | 70 | resprout |
| *Fagus grandifolia* | FAGU.GRA | 250 | 40 | 5 | 1 | 30 | 3000 | 0.5 | 10 | 30 | none |
| *Larix laricina* | LARI.LAR | 150 | 40 | 1 | 1 | 50 | 200 | 0 | 0 | 0 | none |
| *Larix x marschlinsii Coaz, (hybrid)* | LARI.HYB | 80 | 80 | 1 | 1 | 1 | 2 | 0 | 0 | 0 | none |
| *Picea glauca* | PICE.GLA | 200 | 30 | 3 | 2 | 100 | 303 | 0 | 0 | 0 | none |
| *Picea mariana* | PICE.MAR | 200 | 30 | 4 | 2 | 80 | 200 | 0 | 0 | 0 | serotiny |
| *Picea rubens* | PICE.RUB | 300 | 30 | 4 | 1 | 100 | 303 | 0 | 0 | 0 | none |
| *Pinus banksiana* | PINU.BAN | 150 | 20 | 1 | 2 | 30 | 100 | 0 | 0 | 0 | serotiny |
| *Pinus resinosa* | PINU.RES | 200 | 40 | 2 | 3 | 12 | 275 | 0 | 0 | 0 | none |
| *Pinus strobus* | PINU.STR | 300 | 20 | 3 | 3 | 100 | 250 | 0 | 0 | 0 | none |
| *Populus tremuloides* | POPU.TRE | 150 | 20 | 1 | 2 | 1000 | 5000 | 0.9 | 10 | 150 | resprout |
| *Populus spp. (hybrid)* | POPU.HYB | 60 | 60 | 1 | 2 | 1 | 2 | 0.9 | 10 | 60 | resprout |
| *Quercus rubra* | QUER.RUB | 250 | 30 | 3 | 3 | 30 | 3000 | 0.75 | 20 | 200 | resprout |
| *Thuja occidentalis* | THUJ.SPP.ALL | 300 | 30 | 5 | 1 | 45 | 60 | 0.1 | 10 | 60 | none |
| *Tsuga canadensis* | TSUG.CAN | 300 | 60 | 5 | 1 | 30 | 100 | 0 | 0 | 0 | none |

Table 1: Life-history traits parameters of the 19 tree species used in our simulations.

## Details of the prescriptions implemented in Biomass Harvest

We defined 6 types of harvest prescriptions to use with the Biomass Harvest extension of LANDIS-II. Here, we show how they were implemented in the format of the parameters for Biomass Harvest, along with the details of their spatial and temporal distribution controlled through the Magic Harvest extension.

### Control of each prescriptions spatial distribution through the python script

Each prescription was ultimately implemented at each time step through the use of the Magic Harvest extension that we created (4) and a python script called by Magic Harvest. Therefore, all prescriptions had a similar stand ranking, stand qualification and site selection parameters in the format of Biomass Harvest (see below), but these 3 parameters are not representative of how the prescriptions were distributed. Indeed, these parameters were only chosen so that the python script could control in which pixels each prescription would precisely be used. We did this because the Biomass Harvest extension of LANDIS-II was incapable of planting several species in a given forest stand. Controlling prescriptions through the python script thus allowed us to apply different prescriptions in a single stand to plant different species in the different pixels of the stand, which would be necessary for our use of functional planting.

As such, the python script created a raster map of prescriptions at each time step, where numbers 1-19 indicated that a particular prescription be used in this pixel and 0 indicated no prescription. This raster was then indicated as both a management area raster and a stand raster to Biomass Harvest, which was then forced to re-load its parameters. Then, Biomass Succession would harvest 100% of the pixels indicated with the prescription corresponding to the number in the pixels. Here, the stand ranking, stand qualification and site selection parameters that we chose for all of our prescriptions insured that all of the pixels with number 1-19 in the raster map would be harvested without any exception.

For all prescriptions, the python script selected stands by prioritizing them according to a criteria (e.g., those with the most biomass first, or those with the lowest functional diversity first). All prescriptions were applicable only to stands with at least one age cohort of 30 years or older. When a stand was selected for harvesting, the biomass that would be harvested by Biomass Harvest was estimated by a function in the python script, based on the content of the stand. The estimated harvested biomass were summed as stands were selected to be harvested, and the selection stopped when the sum reached the biomass target to harvest with the given prescription for a time step. As such, the biomass harvested by each prescription remained constant through time.

### Clear cuts (CC-PlantFunct, CC-PlantIntens, CC-NormalPlant and CC-NoPlant)

Clear cuts removed all or almost all of the trees present in the stand, and could be followed by the plantation of one or two species in the cell.

#### CC-PlantFunct

CC-PlantFunct was the type of clear cuts used for functional planting. It removed 90% of the biomass of all tree cohorts older than 10 years. As such, it did not remove any age cohort in the pixels of stand, which kept trees of the existing species in place in the pixel. Then, it planted a new age cohort for a given species (see below for the choice of the species). Therefore, we created 17 variation of CC-PlantFunct in Biomass Harvest, one for each species to plant (except the hybrids species, which were not considered useful to the goals of functional planting).

The 17 CC-PlantFunct prescriptions took the following form in the parameters of Biomass Harvest:

Prescription CC-PlantFunct-SPECIES_TO_PLANT

>> STAND RANKING:

StandRanking MaxCohortAge

>> STAND QUALIFICATION FOR CUTTING:

>> None.

>> SITE SELECTION:

SiteSelection Complete

>> COHORT REMOVAL METHOD:

CohortsRemoved SpeciesList

>> Species Cohorts removed

>> ------- ---------------

ABIE.BAL 11-999 (90%)

ACER.RUB 11-999 (90%)

ACER.SAH 11-999 (90%)

BETU.ALL 11-999 (90%)

BETU.PAP 11-999 (90%)

FAGU.GRA 11-999 (90%)

LARI.LAR 11-999 (90%)

LARI.HYB 11-999 (90%)

PICE.GLA 11-999 (90%)

PICE.MAR 11-999 (90%)

PICE.RUB 11-999 (90%)

PINU.BAN 11-999 (90%)

PINU.RES 11-999 (90%)

PINU.STR 11-999 (90%)

POPU.TRE 11-999 (90%)

POPU.HYB 11-999 (90%)

QUER.RUB 11-999 (90%)

THUJ.SPP.ALL 11-999 (90%)

TSUG.CAN 11-999 (90%)

>> PLANTING:

Plant SPECIES_TO_PLANT

The stands affected by CC-PlantFunct were chosen as follow: the functional diversity of every harvestable stands (i.e., not in a protected area) was computed at each time step in the python script that controlled the prescriptions. Then, stands were selected by prioritizing those with the lowest functional diversity. The selected stands had to be located in the extensive zones of the TRIAD zoning system if the prescription was used during a TRIAD scenario.

Once stands were selected, the python script identified which species to plant inside of it for functional planting purposes. To that end, the script identified which functional groups were missing in the stand (i.e., no age cohorts of the species of these groups were present in the cell). If all functional groups were present, the script identified the rarest present group. For each missing functional group (or for the rarest), the script then identified the species that had the highest Probability of Establishment parameter from the Biomass Succession extension for the ecoregion in which the stand was. This ensured that species as adapted as possible to the ecoregion were to be planted. Then, the script randomly applied a CC-PlantFunct prescription for one of the species to plant in one of the pixels of the stand. In this way, the species to plant were randomly planted in the pixels of the stand in equal proportion pixel-wise.

#### CC-PlantIntens

CC-PlantIntens was used to remove all of the present trees in a given stand, and replace them with an even-aged mixed plantation of a hybrid species (hybrid poplar or hybrid larch, depending on the ecoregion) and a commercial species, the white spruce. We therefore created two variation of the prescription for the two hybrid species.

CC-PlantIntens took the following form in the parameters of Biomass Harvest:

Prescription CC-PlantIntens-HYBRID_SPECIES/PICE.GLA

>> STAND RANKING:

StandRanking MaxCohortAge

>> STAND QUALIFICATION FOR CUTTING:

>> None.

>> SITE SELECTION:

SiteSelection Complete

>> COHORT REMOVAL METHOD:

CohortsRemoved SpeciesList

>> Species Cohorts removed

>> ------- ---------------

ABIE.BAL All

ACER.RUB All

ACER.SAH All

BETU.ALL All

BETU.PAP All

FAGU.GRA All

LARI.LAR All

LARI.HYB All

PICE.GLA All

PICE.MAR All

PICE.RUB All

PINU.BAN All

PINU.RES All

PINU.STR All

POPU.TRE All

POPU.HYB All

QUER.RUB All

THUJ.SPP.ALL All

TSUG.CAN All

>> PLANTING:

Plant HYBRID_SPECIES PICE.GLA

CC-PlantIntens was only used in the intensive zones of the TRIAD scenarios, in order to simulate an intensive form of forestry focused on wood production alone as is typical of the TRIAD zoning system. Stands to harvest were selected by prioritizing those with the largest amount of biomass. CC-PlantIntens was then applied uniformly to all pixels in the selected stands.

#### CC-NormalPlant

The goal of CC-NormalPlant was to imitate the fact that a plantation of species present before a clear cut (often commercial species) are regularly done in Quebec to help the regeneration of the stand (5).

The CC-NormalPlant prescription took the same form as CC-FunctPlant in the parameter files of Biomass Harvest (see above). It selected stands by prioritizing those with the largest amount of biomass in the area where it was applied. Once stands were selected, the python script identified the two dominant species in the stand in terms of biomass. These two dominant species were then re-planted after the cut by randomly assigning their planting in the pixels of the stands. However, based on expert opinions, we made sure that the basalm fir was never replanted, even if it was dominant in the stand before the cut. Instead, it was replaced by either white or black spruce, depending on the other dominant species in the stand. This was done as the basalm fir is often seen as an undesirable tree species by the industry that is often too present in the regenerating stands, and thus not replanted.

#### CC-NoPlant

CC-NoPlant was the simplest form of clear cut. It selected stands by prioritizing those with the largest amount of biomass, and applied uniformly to all pixels of the selected stands. It took the following form in the parameter file of Biomass Harvest:

Prescription CPRS-NOPLANT

>> STAND RANKING:

StandRanking MaxCohortAge

>> STAND QUALIFICATION FOR CUTTING:

>> None.

>> SITE SELECTION:

SiteSelection Complete

>> COHORT REMOVAL METHOD:

CohortsRemoved SpeciesList

>> Species Cohorts removed

>> ------- ---------------

ABIE.BAL 11-999 (90%)

ACER.RUB 11-999 (90%)

ACER.SAH 11-999 (90%)

BETU.ALL 11-999 (90%)

BETU.PAP 11-999 (90%)

FAGU.GRA 11-999 (90%)

LARI.LAR 11-999 (90%)

LARI.HYB 11-999 (90%)

PICE.GLA 11-999 (90%)

PICE.MAR 11-999 (90%)

PICE.RUB 11-999 (90%)

PINU.BAN 11-999 (90%)

PINU.RES 11-999 (90%)

PINU.STR 11-999 (90%)

POPU.TRE 11-999 (90%)

POPU.HYB 11-999 (90%)

QUER.RUB 11-999 (90%)

THUJ.SPP.ALL 11-999 (90%)

TSUG.CAN 11-999 (90%)

### Selection cuts (SC)

Selection cuts were implemented to imitate an irregular shelterwood repeated other multiple years in the same stand as a form of uneven-aged management often practised in Quebec. It harvested 30% of the biomass of all age cohorts of 30 years of age or older, and applied every 30 years to the same stand for 90 years after its first application in the stand.

SC took the following form in the parameter files of Biomass Harvest:

Prescription SC

>> STAND RANKING:

StandRanking MaxCohortAge

>> STAND QUALIFICATION FOR CUTTING:

>> None.

>> SITE SELECTION:

SiteSelection Complete

>> COHORT REMOVAL METHOD:

CohortsRemoved SpeciesList

>> Species Cohorts removed

>> ------- ---------------

ABIE.BAL 30-999(30%)

ACER.RUB 30-999(30%)

ACER.SAH 30-999(30%)

BETU.ALL 30-999(30%)

BETU.PAP 30-999(30%)

FAGU.GRA 30-999(30%)

LARI.LAR 30-999(30%)

LARI.HYB 30-999(30%)

PICE.GLA 30-999(30%)

PICE.MAR 30-999(30%)

PICE.RUB 30-999(30%)

PINU.BAN 30-999(30%)

PINU.RES 30-999(30%)

PINU.STR 30-999(30%)

POPU.TRE 30-999(30%)

POPU.HYB 30-999(30%)

QUER.RUB 30-999(30%)

THUJ.SPP.ALL 30-999(30%)

TSUG.CAN 30-999(30%)

SC prioritized stands with the most biomass. New stands to harvest with SC were selected if the biomass target to harvest with SC at each time step wasn't reached by harvesting the stands selected in previous years for harvesting with SC, to which the repeated prescriptions of SC applied every 30 years. Once a stand was selected to be harvested with SC for the next 90 years, it became unavailable to other prescriptions until the end of those 90 years. SC applied uniformly to every pixels in a stand and was not followed by any plantation. In TRIAD scenarios, SC was only applied to stands contained in the extensive zones.

### Commercial Thinning (CT)

Commercial Thinning was implemented to imitate its current use in Quebec. As such, it harvested a large amount of biomass from younger age cohorts in a stand, and a smaller amount for older cohorts. It was used with two variations: an "intensive" and a "non-intensive" form. The intensive form was used in the intensive areas of the TRIAD scenarios, while the non-intensive form was used in the BAU scenarios.

CT was implemented in the following way in the parameter file of Biomass Harvest:

Prescription Thinning

>> STAND RANKING:

StandRanking MaxCohortAge

>> STAND QUALIFICATION FOR CUTTING:

>> None.

>> SITE SELECTION:

SiteSelection Complete

>> COHORT REMOVAL METHOD:

CohortsRemoved SpeciesList

>> Species Cohorts removed

>> ------- ---------------

ABIE.BAL 1-30 (80%) 31-50 (66%) 51-70 (60%) 71-90 (60%) 91-100 (40%) 101-120 (5%)

ACER.RUB 1-30 (80%) 31-50 (66%) 51-70 (60%) 71-90 (60%) 91-100 (40%) 101-120 (5%)

ACER.SAH 1-30 (80%) 31-50 (66%) 51-70 (60%) 71-90 (60%) 91-100 (40%) 101-120 (5%)

BETU.ALL 1-30 (80%) 31-50 (66%) 51-70 (60%) 71-90 (60%) 91-100 (40%) 101-120 (5%)

BETU.PAP 1-30 (80%) 31-50 (66%) 51-70 (60%) 71-90 (60%) 91-100 (40%) 101-120 (5%)

FAGU.GRA 1-30 (80%) 31-50 (66%) 51-70 (60%) 71-90 (60%) 91-100 (40%) 101-120 (5%)

LARI.LAR 1-30 (80%) 31-50 (66%) 51-70 (60%) 71-90 (60%) 91-100 (40%) 101-120 (5%)

LARI.HYB 1-30 (80%) 31-50 (66%) 51-70 (60%) 71-90 (60%) 91-100 (40%) 101-120 (5%)

PICE.GLA 1-30 (80%) 31-50 (66%) 51-70 (60%) 71-90 (60%) 91-100 (40%) 101-120 (5%)

PICE.MAR 1-30 (80%) 31-50 (66%) 51-70 (60%) 71-90 (60%) 91-100 (40%) 101-120 (5%)

PICE.RUB 1-30 (80%) 31-50 (66%) 51-70 (60%) 71-90 (60%) 91-100 (40%) 101-120 (5%)

PINU.BAN 1-30 (80%) 31-50 (66%) 51-70 (60%) 71-90 (60%) 91-100 (40%) 101-120 (5%)

PINU.RES 1-30 (80%) 31-50 (66%) 51-70 (60%) 71-90 (60%) 91-100 (40%) 101-120 (5%)

PINU.STR 1-30 (80%) 31-50 (66%) 51-70 (60%) 71-90 (60%) 91-100 (40%) 101-120 (5%)

POPU.TRE 1-30 (80%) 31-50 (66%) 51-70 (60%) 71-90 (60%) 91-100 (40%) 101-120 (5%)

POPU.HYB 1-30 (80%) 31-50 (66%) 51-70 (60%) 71-90 (60%) 91-100 (40%) 101-120 (5%)

QUER.RUB 1-30 (80%) 31-50 (66%) 51-70 (60%) 71-90 (60%) 91-100 (40%) 101-120 (5%)

THUJ.SPP.ALL 1-30 (80%) 31-50 (66%) 51-70 (60%) 71-90 (60%) 91-100 (40%) 101-120 (5%)

TSUG.CAN 1-30 (80%) 31-50 (66%) 51-70 (60%) 71-90 (60%) 91-100 (40%) 101-120 (5%)

CT prioritized stands with the largest biomass. In its intensive form, CT was applied 3 time to a given stand, with a 30 years period between application. As for SC, new stands were added to the intensive CT if the target to harvest with CT for the time step wasn't reached by the repeated CT at this time step. Once intensive CT was applied to a stand, the stand became unavailable to other prescriptions until the end of its 60 years of repeated application. In its non-intensive form, CT was only applied one time, with no repeated application. CT applied uniformly to every pixels in a stand and was not followed by any plantation.

## Details of the catastrophic events – Distribution and effects

The "catastrophic" disturbance events that we triggered at t = 100 in most of our simulations were simulated as harvest prescriptions by the use of Biomass Harvest and Magic Harvest (see section B for more). In this way, we were able to precisely control where and when these events triggered, as well as their impacts on the forest stands. Here, we explain how their distribution and effects were defined.

### Large Fire

The large fire touched 70% of the pixel of forests of the landscape, with the 30% remaining being left untouched as fire refugias (see main article). To that end, every stand was put into the list of stands impacted by the fire, and fire refugias were progressively created by removing stands from the list. This process is done in the python script magicHarvestFunctions_v3.py available in the files associated to the article (see function "megaFireCatastrophy"). We represent it here in pseudocode:

while surfaceOfRefugias < 0.3 * surfaceOfForests:

# We take a number from a power law distribution

# Going from 1 to 100, with an alpha parameter of 0.07

# Will generate a lot of small values close to 1, and a few large values

surfaceOfNextRefugia = powerLawDistributionRandomNumber(1, 100, 0.07)

# Next, we choose a random stand and propagate the refugia

# from neighbouring stand to neighbouring stand until the surface

# (in hectares) chosen with the power law is reached.

standToStartRefugia = chooseARandomStand(listOfStandsID)

listOfStandsInRefugia = list(standToStartRefugia)

while area(listOfStandsInRefugia) < surfaceOfNextRefugia:

propagateRefugia(listOfstandsInRefugia)

Once the refugias had been defined in that way, special harvest prescriptions were applied to all of the other (burned) stands. These prescriptions were made so that the loss of biomass for each age cohort changed according to the species, with fire tolerant species losing less biomass. This is easily made in Biomass Harvest, with the prescriptions imitating the effects of the large fire taking the following form:

Prescription MegaFire-100%Effect

>> STAND RANKING:

StandRanking MaxCohortAge

>> STAND QUALIFICATION FOR CUTTING:

>> None.

>> SITE SELECTION:

SiteSelection Complete

>> COHORT REMOVAL METHOD:

CohortsRemoved SpeciesList

>> Species Cohorts removed

>> ------- ---------------

ABIE.BAL 1-999 (100%)

ACER.RUB 1-999 (90%)

ACER.SAH 1-999 (90%)

BETU.ALL 1-999 (100%)

BETU.PAP 1-999 (100%)

FAGU.GRA 1-999 (100%)

LARI.LAR 1-999 (100%)

LARI.HYB 1-999 (100%)

PICE.GLA 1-999 (90%)

PICE.MAR 1-999 (90%)

PICE.RUB 1-999 (100%)

PINU.BAN 1-999 (90%)

PINU.RES 1-999 (80%)

PINU.STR 1-999 (80%)

POPU.TRE 1-999 (90%)

POPU.HYB 1-999 (90%)

QUER.RUB 1-999 (80%)

THUJ.SPP.ALL 1-999 (100%)

TSUG.CAN 1-999 (100%)

However, as indicated in the main text of the article, we further varied the effect of the fire from stand to stand according to the Community Weighted Mean (CWM) of the fire tolerance traits for all tree species in the stand, based on the biomass of their respective age cohorts. As such, we further created 5 prescriptions for the large fire, each of them further reducing the biomass lost for all species as indicated in Table 2 of the main article. As an example, here is the prescription for stands suffering only 90% of the "full" effect of the large fire (as indicated in the prescription above):

Prescription MegaFire-90%Effect

>> STAND RANKING:

StandRanking MaxCohortAge

>> STAND QUALIFICATION FOR CUTTING:

>> None.

>> SITE SELECTION:

SiteSelection Complete

>> COHORT REMOVAL METHOD:

CohortsRemoved SpeciesList

>> Species Cohorts removed

>> ------- ---------------

ABIE.BAL 1-999 (90%)

ACER.RUB 1-999 (81%)

ACER.SAH 1-999 (81%)

BETU.ALL 1-999 (90%)

BETU.PAP 1-999 (90%)

FAGU.GRA 1-999 (90%)

LARI.LAR 1-999 (90%)

LARI.HYB 1-999 (90%)

PICE.GLA 1-999 (81%)

PICE.MAR 1-999 (81%)

PICE.RUB 1-999 (90%)

PINU.BAN 1-999 (81%)

PINU.RES 1-999 (72%)

PINU.STR 1-999 (72%)

POPU.TRE 1-999 (81%)

POPU.HYB 1-999 (81%)

QUER.RUB 1-999 (72%)

THUJ.SPP.ALL 1-999 (90%)

TSUG.CAN 1-999 (90%)

As indicated in the main article, the relation between the age-cohort level and stand-level values of biomass loss and the fire tolerance trait of the species and of the stand were chosen arbitrarily, based on expert opinion.

### Severe Drought

The severe drought was implemented in a way similar to the large fire. However, it impacted every stand of the landscape, with no refugias being created. The age-cohort level and stand-level effects were dealt with in the same way than with the large fire (see above).

### Mountain Pine Beetle epidemic

The Mountain Pine Beetle (MPB) epidemic impacted only the stands of the landscape that contained one of its potential host species: the jack pine (*Pinus banksiana*), the eastern white pine (*Pinus strobus*) and the red pine (*Pinus resinosa*). All age-cohorts of these three species lost 80% of their biomass across the entire landscape, with the other species left untouched. However, this effect changed from stand to stand according to their ratio of biomass between the host species and non-host species. We based the modulation of this effect on the data from the Figure 1b of Jactel, Moreira, and Castagneyrol (6). The figure shows the log response ratio of tree diversity (i.e., if the stand studied is "mixed" or "pure" as to the hosts species of the MPB) on the abundance or damages of borer insects among 45 studies. The mean of this log ratio was around -0.4557, which can be interpreted in the way that "mixed" stands had only 57% of the abundance of borer insects or damage from these insects as compared to "pure" stands. We implemented this information in the following way: Pure stands (with 100% - 90% of their biomass composed of the three host species) suffered 100% of the impact of the MPB in our simulations (i.e., 80% reduction in the biomass of the cohort of each host species). In contrast, stands with 50—40% of their biomass being composed of non-host species suffered 57% of the MPB (based on the log ratio of 6). The remaining 8 categories of stands that we defined according to the ratio of host species (90 – 80%, 80% - 70%, 70 – 60%, 60 – 50%, 40 – 30%, etc.) had a MPB impact that varied according to a linear relation defined by the two previous categories (100% effect for 100 – 90% host abundance, 57% effect for 50 – 40% host abundance). As such, the effect for each category was defined by the equation:

$$MPBeffectforcategory=0.86*Hostabundanceoftheupperboundofthecategory+0.14$$

This variation of effect was finally implemented through 10 different prescriptions defined in the Biomass Harvest parameter file, in a way similar to the large fire and severe drought (see above).

## Clustering to create the functional groups

We clustered our 17 species (without taking into account the two hybrid species) into 5 functional groups based on a database of 10 functional traits. We chose traits related to the resistance, resilience and growth of trees facing a disturbance: post fire regeneration (PFR), seed mass (SM), specific leaf area (SLA), wood density (WD), leaf nitrogen content (Nmass), bark thickness coefficient (BTcoef), maximum height (maxH), fire tolerance (FirT), drought tolerance (DT) and shade tolerance (ST). Values for each trait came from (7) and from the core parameters that we used in LANDIS-II (see sections above). As we wanted the clustering to produce biologically meaningful functional groups (meaning groups that displayed species of different biological categories or life strategies), we added the characteristic of being an angiosperm or a gymnosperm as an additional trait for the clustering.

Having chosen our traits, we calculated a dissimilarity matrix based on our traits using the Gower's distance between each species (8). During this calculation, we ponderated the different traits according to the correlation between them, with correlated/redondant traits being ponderated less. The correlation between traits was assessed by a statistical test using Spearman’s rank-order correlation (ρ). When x traits were found to be significantly correlated together, we ponderated them with the value 1/x. Ponderating the traits in that way allowed us to reduce the importance of a redundant information contained in the correlated traits during the following clustering. In the end, the SLA, WD and Nmass traits were found to be correlated together, and FirT and BTcoef with one another. This resulted in a ponderation of 0.33 for SLA, WD and Nmass, and a ponderation of 0.5 for FirT and BTcoeff. The correlation between traits is shown in Figure 1.


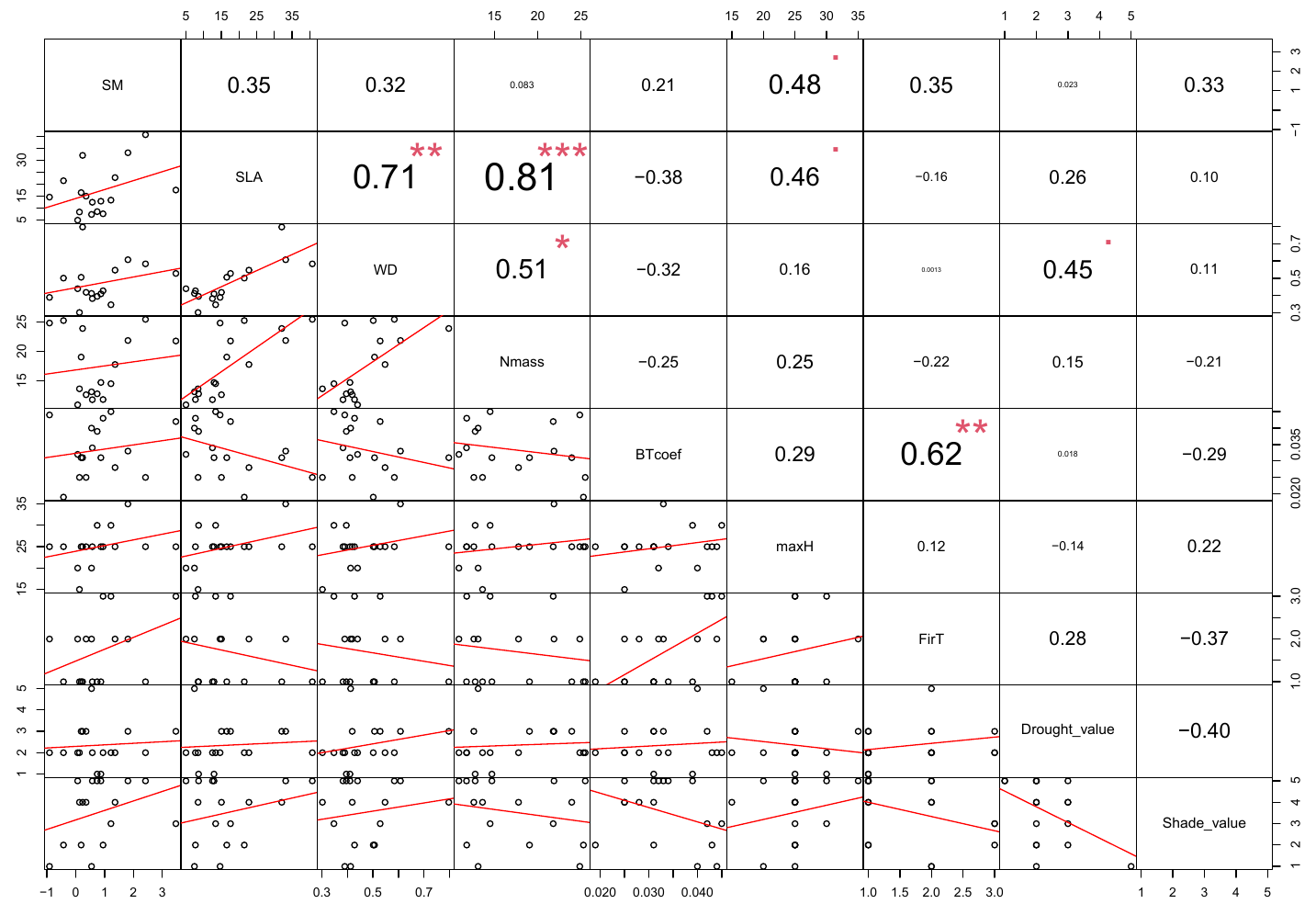
Figure 1: Correlogram of the trait values for our 17 different species to cluster in functional groups. Only numerical traits are represented.

With the dissimilarity matrix calculated, we performed a hierarchical clustering with Ward's method to aggregate species with smaller distances between them into functional groups. We then validated the number of functional groups through the analysis of the silhouette width. The dendrogram of the clustering is given below, with the final functional groups kept indicated by the blue envelopes in Figure 2.


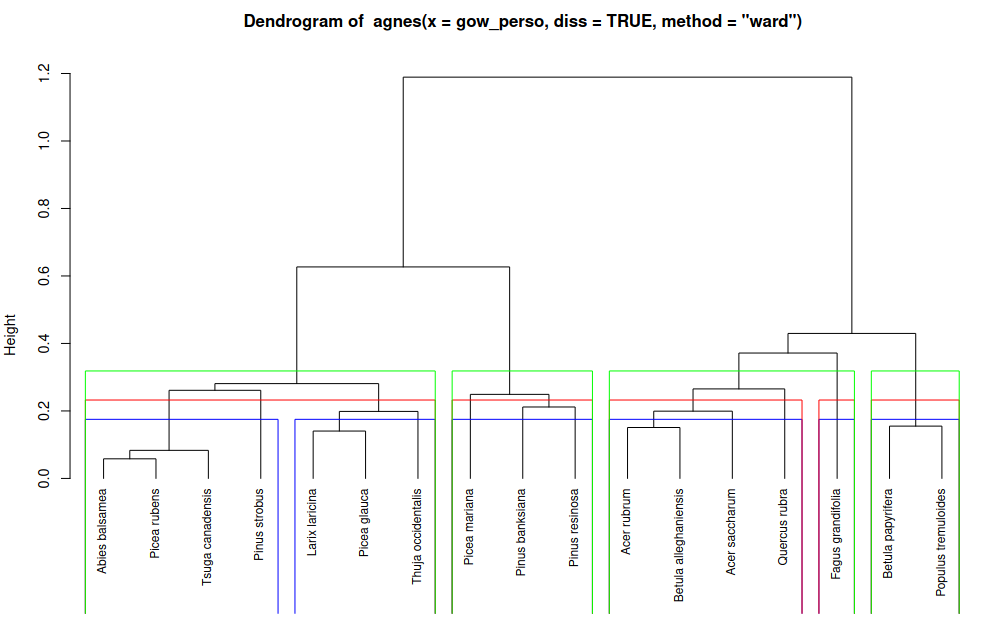
Figure 2: Dendrogram showing the clustering of our 17 species of intesrest into 5 functional groups (blue envellopes). Note that Fagus grandifolia has been included in the last group on the right of the dendrogram, with Betulla papyrifera and Populus tremuloides (see text).

The final 5 functional groups were numbered 1 to 5 (from left to right on the dendrogram), and interpreted in the following way:

1 – Softwood trees with high tolerance to shade

2 – Softwood trees with low tolerance to shade and low resistance to fire

3 – Softwood trees with low tolerance to shade and high resistance to fire

4 – Late succession hardwoods trees

5 – Pionner hardwood trees

As Fagus grandifolia was between group 4 and 5, we decided to include it in group 5 as it is often found as a pionner species.

The resulting radar plots for the mean value of each trait for the different groups are shown in Figure 3.


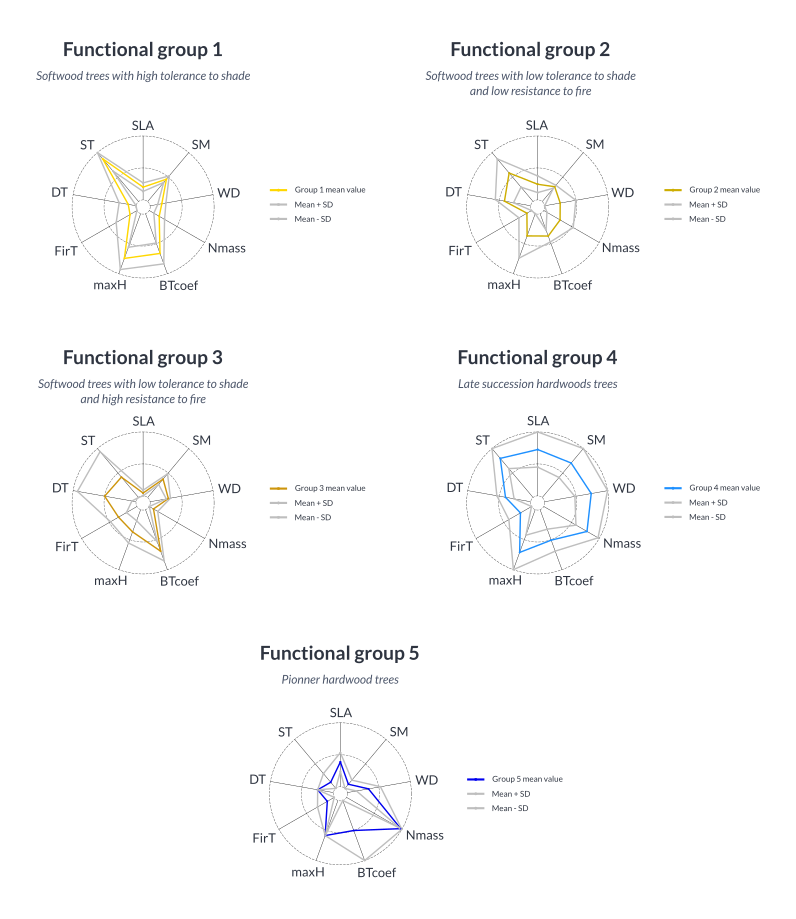
Figure 3: Radar plots of the mean values and standard deviation for the 5 functional groups resulting from the clustering, and for the 9 quantitative traits in our database.

## Surface burned in the different scenarios

As stated in the main text of the article, the forest fires simulated by the Base Fire extension of LANDIS-II were stochastic in nature. As such, the surface burned at every time step presented a large amount of variability between replicates and scenarios. In addition, the surface burned tended to increase through time in scenarios with climate change (RCP 4.5 and RCP 8.5). Moreover, the north of our simulated area tended to have much more forest fires (and more surface burned) as it was situated in a different homogeneous fire zone (as defined by 9) than the south of the area. Consequently, the south of the area had less surface burned by time step when compared to the north (by a factor of almost ten). We display these tendencies in Figure 4, Figure 5 and Figure 6, which show the surface burned in the whole of the landscape, the north and the south respectively. Keep in mind that all of these figure only count the surface burned by the fires created by the Base Fire extension, without taking into account the "Large Fire" which was one of the three catastrophic events (and which was simulated with the Biomass Harvest and Magic Harvest extensions; see section C).


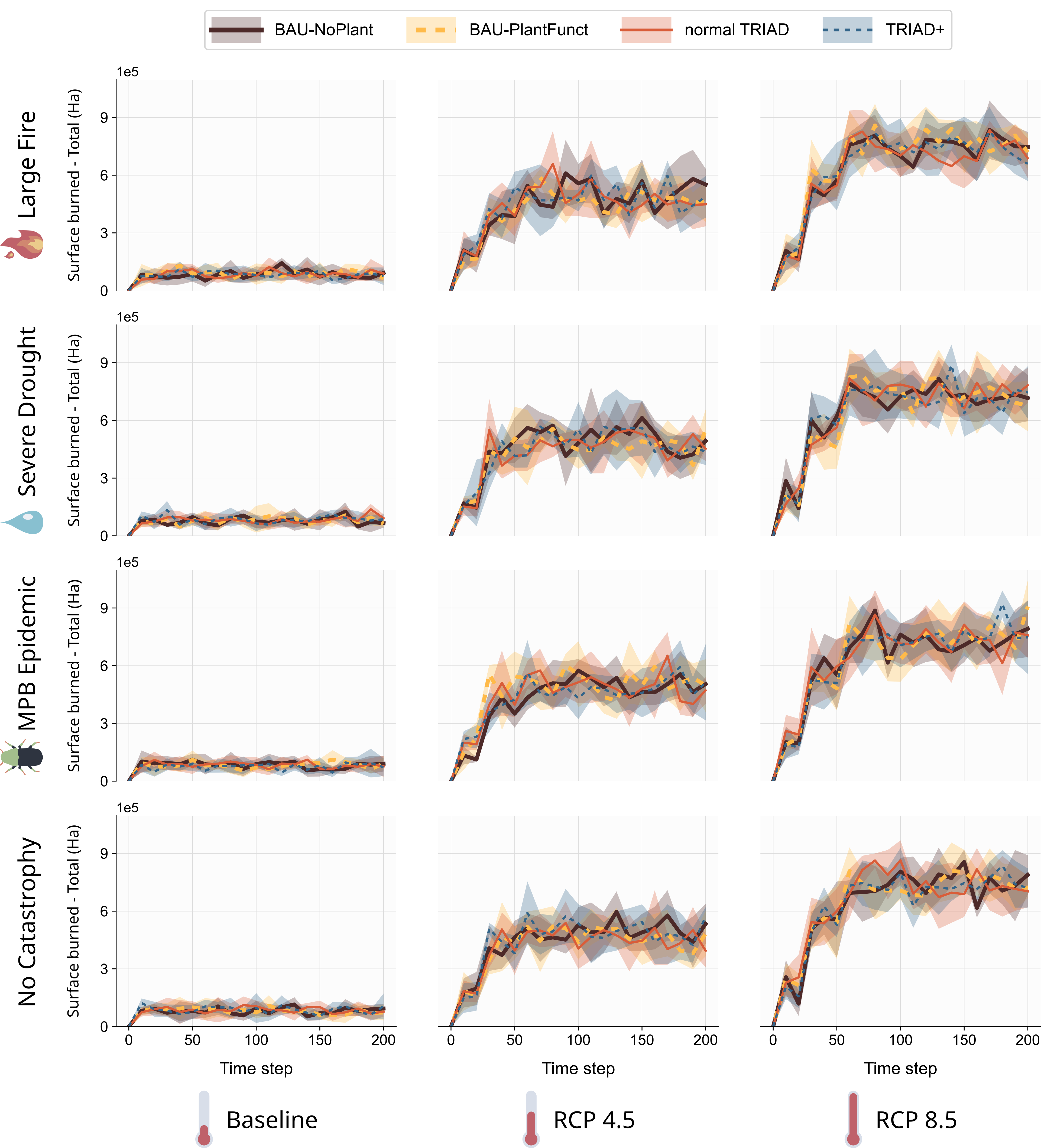
Figure 4: Temporal variation of the surface burned by the fires made with the Base Fire extensions (thus excluding the "Large Fire" catastrophic event) in the whole of the landscape for each combination of management, climate and catastrophe scenario. Solid lines are mean values and envelops are standard deviation across 5 simulation replicates.


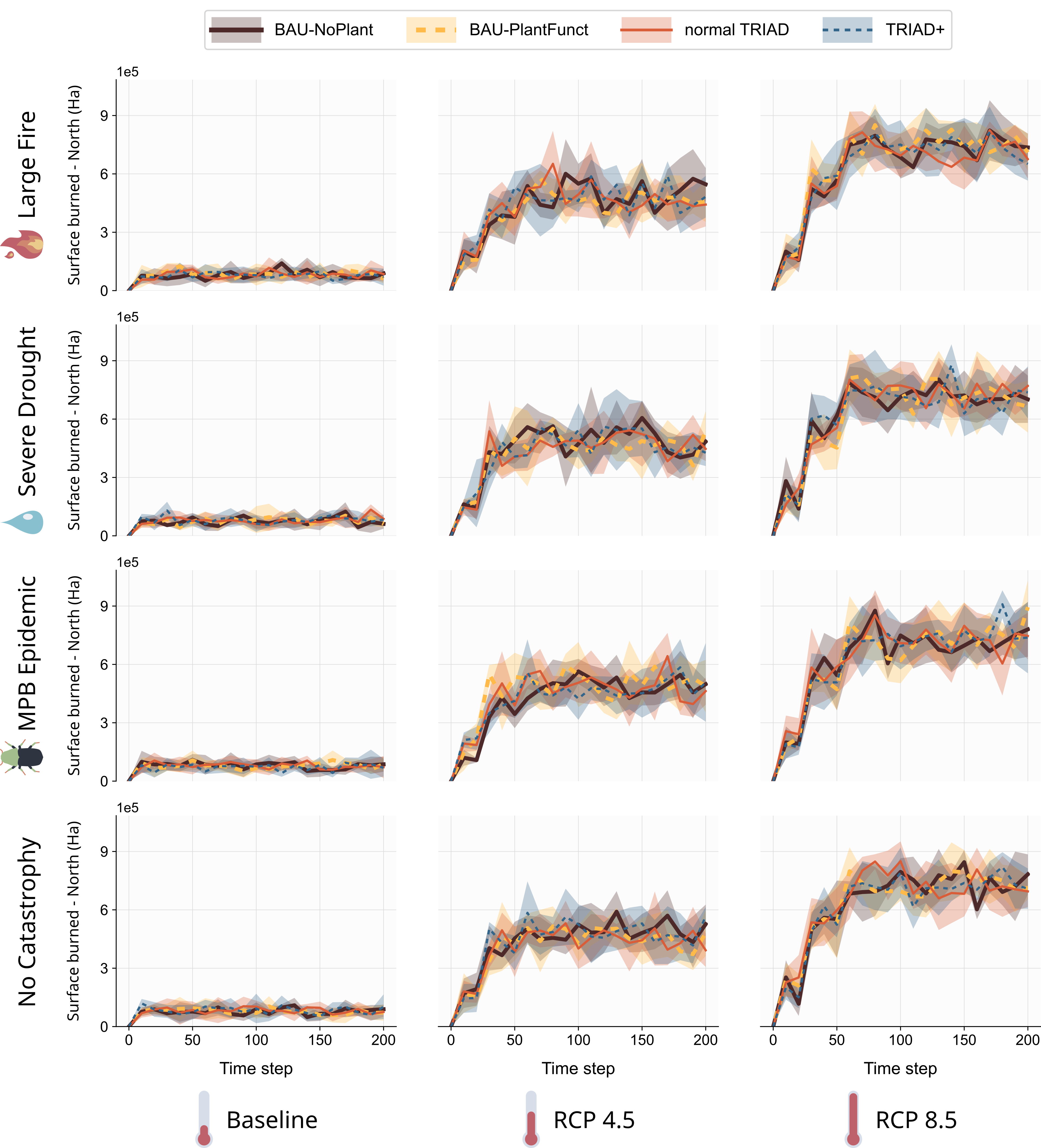


Figure 5: Temporal variation of the surface burned by the fires made with the Base Fire extensions (thus excluding the "Large Fire" catastrophic event) in the north of the landscape for each combination of management, climate and catastrophe scenario. Solid lines are mean values and envelops are standard deviation across 5 simulation replicates.


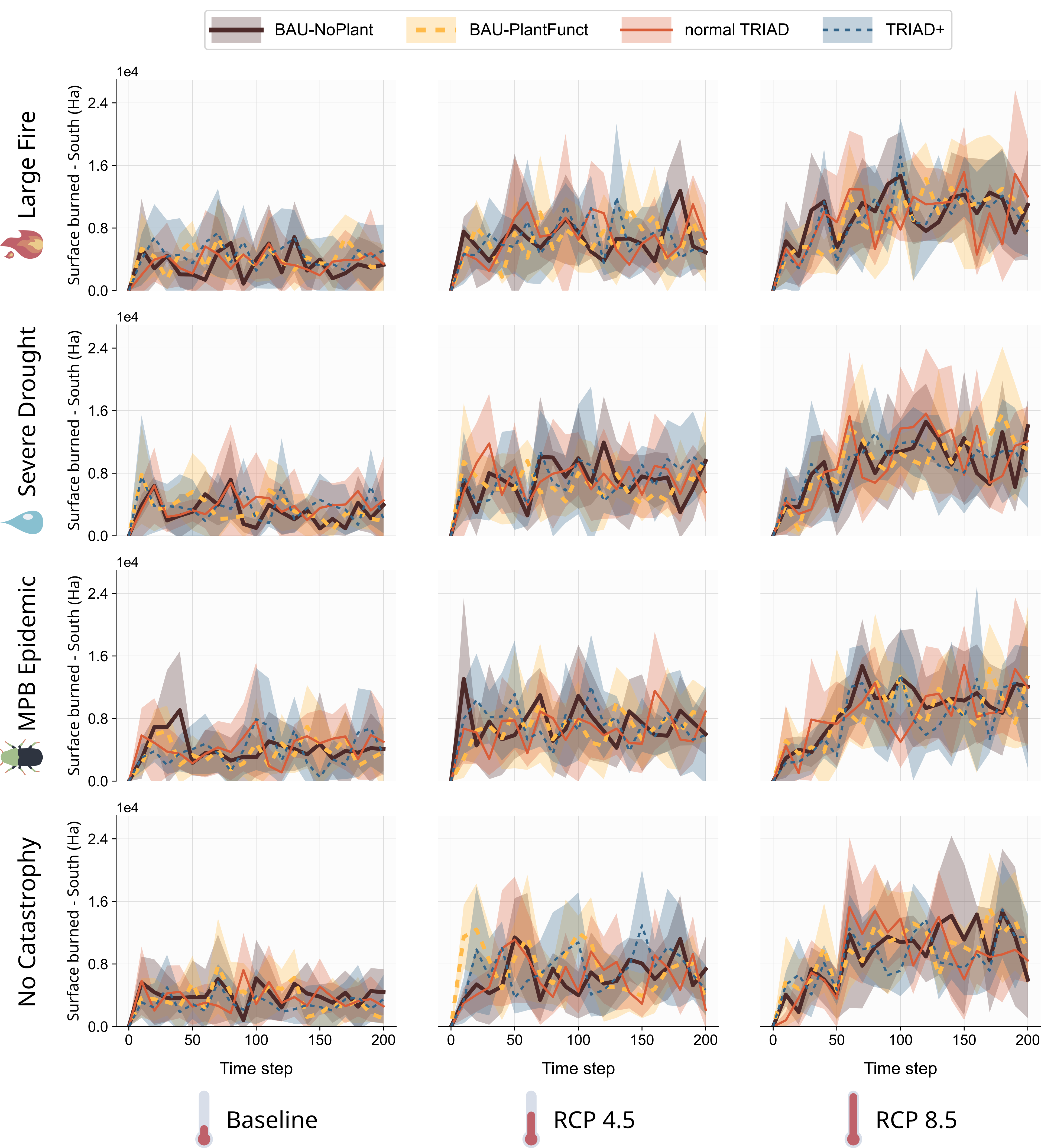


Figure 6: Temporal variation of the surface burned by the fires made with the Base Fire extensions (thus excluding the "Large Fire" catastrophic event) in the south of the landscape for each combination of management, climate and catastrophe scenario. Solid lines are mean values and envelops are standard deviation across 5 simulation replicates.

## Evaluating the size of the differences in the net change of the different management scenarios

In the article, we show that the TRIAD+ and BAU-PlantFunct scenario tended to improve the resilience of the mature biomass of stands impacted by a catastrophic event according to three different resilience measures. However, it can be difficult to convey the practical importance of these improvement for management decisions. In order to give a sense of scale to these differences, we propose an additional figure (Figure 7) showing two types of values side by side. The first is the raw net change (in Mg) of the sum of the mature biomass of all forest stands impacted by a catastrophic event, varying from scenario to scenario. This is slightly different than the net change value described in Figure 7 of the main article, which is a relative net change (in % of the value of mature biomass in the stand before the catastrophic event). In addition, Figure 7 of the main article shows a relative net change at the stand-scale, with bars representing the mean value for all stands, while we show here a sum across all stands. The second value shown on this additional figure is the biomass target to harvest at each time step across all scenarios. In this way, we can compare the magnitude of the difference in the biomass “preserved” after the catastrophic event by improvement in forest resilience in some management scenarios to the practical reality of the biomass harvested regularly in the landscape. As shown in Figure 7, the differences between scenario are small compared to the target biomass to harvest. This suggests that while the TRIAD+ and BAU-PlantFunct scenarios are able to increase forest resilience and to help the landscape recover more biomass than the normal TRIAD and BAU-NoPlant scenarios, their effect is not large enough to have a high influence on management decisions.


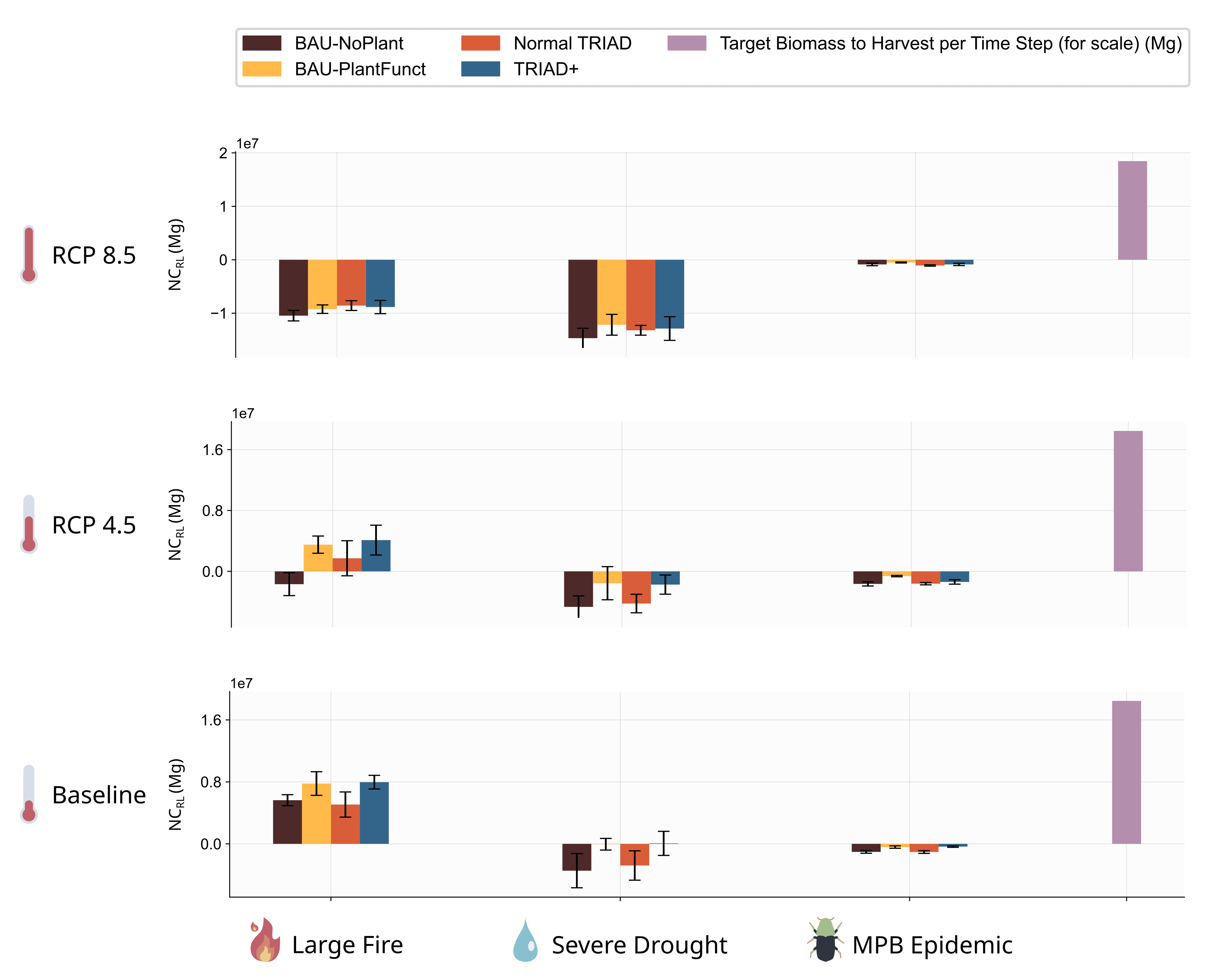
Figure 7: Bar plots showing the raw net change of the mature biomass of all stands impacted by a catastrophic event (NC_RL_). Each colored bar represents the mean and the black vertical lines the standard deviation over the 5 simulation replicates for a given combination of climate, management strategy and catastrophic event. The purple bars on the right-hand side represent the target biomass to harvest per time step in all of the simulations, independent of the management strategy used. It gives a sense of scale as to how much mature biomass can be "protected" by a given management strategy by increasing the resilience of the stands of the landscape.

## Differences in the dynamic of the mature biomass in the north and in the south of our study area

Our study area was divided into two fire regions, with the northern region (being in the boreal forest biome) having much more fires than the south (being in the mixed/temperate forest biome). (see main text of the article and the supplemental material of 10). Figure 8 and Figure 9 display the dynamic of the mature biomass of the landscape in these two regions, showing that differences between climate scenarios are much more marked in the north than in the south. This is due to the fact that our climate scenarios also influenced the fire regime in both regions, with fires becoming increasingly more frequent in the RCP 4.5 and 8.5 scenarios (see text of the main article). This effect of the climate on forest fires was particularly strong in the north of our area, leading to the forests that they contained being affected by frequent fires (through the Base Fire extension) and altered tree growth (through the Biomass Succession extension).


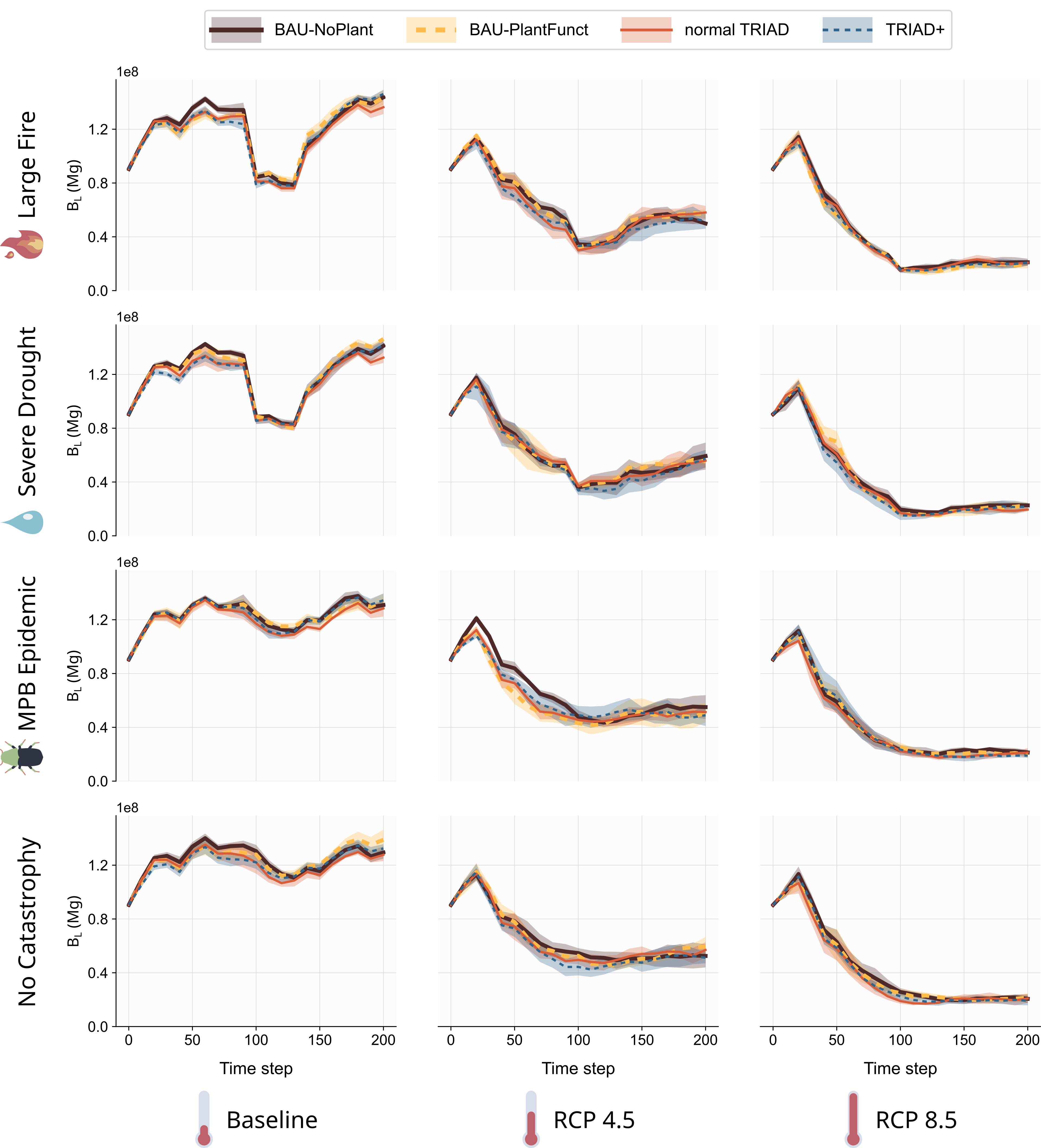


Figure 8: Temporal variation of the Total Mature Biomass BL in the north of the landscape (defined by the northern fire region) for each combination of management, climate and catastrophe scenario. Solid lines are mean values and envelops are standard deviation across 5 simulation replicates


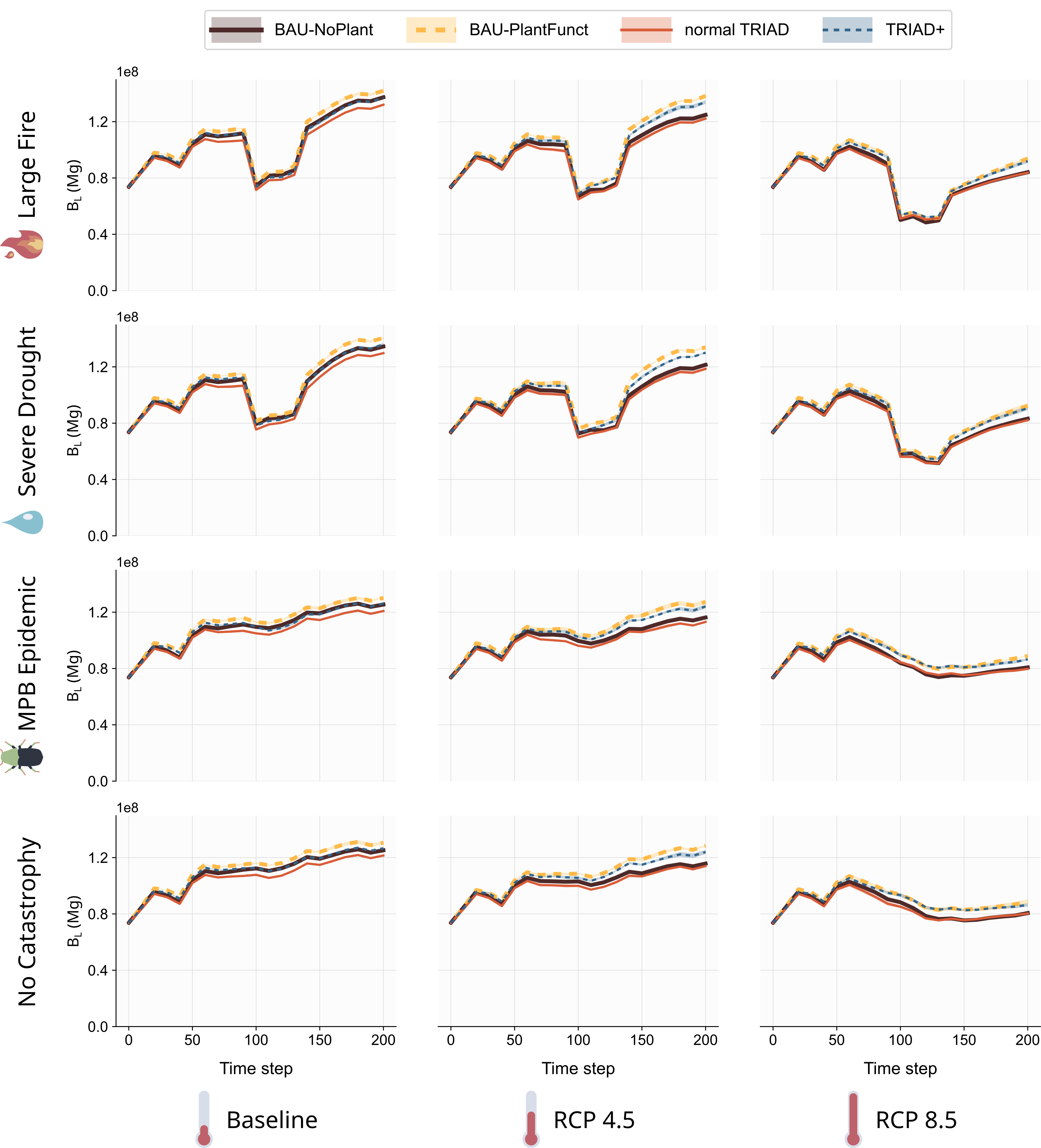


Figure 9: Temporal variation of the Total Mature Biomass BL in the south of the landscape (defined by the southern fire region) for each combination of management, climate and catastrophe scenario. Solid lines are mean values and envelops are standard deviation across 5 simulation replicates

References

1. Tremblay JA, Boulanger Y, Cyr D, Taylor AR, Price DT, St-Laurent MH. Harvesting interacts with climate change to affect future habitat quality of a focal species in eastern Canada’s boreal forest. Yang J, editor. PLOS ONE. 2018 Feb 7;13(2):e0191645.

2. Farrar J. Trees in Canada. Published by Fitzhenry & Whiteside Ltd. and Canadian Forest Service. Natural Resources Canada, in cooperation with the Canada Communication Group—Publishing, Supply and Services Canada. 1995;

3. Burns RM, Honkala BH, others. Silvics of North America: 1. Conifers; 2. Hardwoods Agriculture Handbook 654. US Department of Agriculture, Forest Service, Washington, DC. 1990;

4. Hardy C. Magic Harvest LANDIS-II extension [Internet]. 2022. Available from: https://github.com/Klemet/LANDIS-II-Magic-Harvest

5. Bureau du forestier en chef du Quebec/ Quebec’s Office of the Chief Forester. Manuel de détermination des possibilités forestières, 2013-2018. Roberval, Québec: Bureau du forestier en chef; 2013.

6. Jactel H, Moreira X, Castagneyrol B. Tree Diversity and Forest Resistance to Insect Pests: Patterns, Mechanisms, and Prospects. Annu Rev Entomol. 2021 Jan 7;66(1):277–96.

7. Paquette A, Messier C. The effect of biodiversity on tree productivity: from temperate to boreal forests. Global Ecology and Biogeography. 2011;20(1):170–80.

8. Gower JC. A general coefficient of similarity and some of its properties. Biometrics. 1971;857–71.

9. Boulanger Y, Gauthier S, Burton PJ. A refinement of models projecting future Canadian fire regimes using homogeneous fire regime zones. Canadian Journal of Forest Research. 2014 Apr;44(4):365–76.

10. Hardy C, Messier C, Boulanger Y, Cyr D, Filotas É. Land sparing and sharing patterns in forestry: exploring even-aged and uneven-aged management at the landscape scale. Landsc Ecol. 2023 Nov 1;38(11):2815–38.
