# Supplementary material for: Climate is stronger than you think: Exploring functional planting and TRIAD zoning for increased forest resilience to extreme disturbances
Source: PLoS One. 2025 Jun 27;20(6):e0326627. doi: 10.1371/journal.pone.0326627 (PMC12204526; doi:10.1371/journal.pone.0326627)
Supplement: Attachment — Submitted filename: Response To Reviewers.docx [file pone.0326627.s003.docx]

Response to Reviewer's Comments on submission PONE-D-24-22896

Climate is stronger than you think: Exploring functional planting and TRIAD zoning for increased forest resilience to extreme disturbances

Clément Hardy^1*^, Christian Messier^1,5^, Yan Boulanger^2^, Dominic Cyr^3^, Élise Filotas^4^

^1^Department of Biological Sciences, Centre d’Étude de la Forêt (CEF), Université du Québec à Montréal, Canada (UQAM)

^2^Natural Resources Canada, Canadian Forest Service, Laurentian Forestry Centre, Québec, QC, Canada

^3^Sciences et Technology Branch, Environment and Climate Change Canada, Gatineau, QC, Canada

^4^Department of Science and Technology, Université du Québec (TELUQ), Canada
^5^Department of Natural Sciences, Institut des Sciences de la forêt tempérée (ISFORT), Université du Québec en Outaouais (UQO)

*Corresponding author: [clem.hardy@outlook.fr](mailto:clem.hardy@outlook.fr) (CH)

We thank the reviewers and editors for their evaluation of our manuscript and their detailed comments, which helped improve the quality of the manuscript. In the following document, we provide a detailed response to each comment along with a description of the changes made to the manuscript.

# 1. Journal requirements

**Comment 1 – Journal requirements:**

Please ensure that your manuscript meets PLOS ONE's style requirements, including those for file naming. The PLOS ONE style templates can be found at

<https://journals.plos.org/plosone/s/file?id=wjVg/PLOSOne_formatting_sample_main_body.pdf>

and

<https://journals.plos.org/plosone/s/file?id=ba62/PLOSOne_formatting_sample_title_authors_affiliations.pdf>

**Response:**

We thank the editor for their evaluation, and the precious comments that they have given us to improve the manuscript.

Following this first comment, we have re-formatted several parts of the manuscript to fit PLOS ONE’s style requirements by editing the style of headers, changing citations of figures and tables, putting the figures in the right format (individual .tiff files), and putting tables inside the manuscript. We have also changed the formatting of authors affiliation as required by the guidelines provided.

**Comment 2 – Journal requirements:**

Please note that PLOS ONE has specific guidelines on code sharing for submissions in which author-generated code underpins the findings in the manuscript. In these cases, we expect all author-generated code to be made available without restrictions upon publication of the work. Please review our guidelines at https://journals.plos.org/plosone/s/materials-and-software-sharing#loc-sharing-code and ensure that your code is shared in a way that follows best practice and facilitates reproducibility and reuse.

**Response:**

The Figshare repository linked in the “Data availability” section of our manuscript (<https://figshare.com/s/8a7e11afb70e3340a4f4>) contains all of the code necessary to generate the input files of our simulations, run the simulations, and generate the figures. The code is currently available without restriction with the link provided and will become publicly referenced thanks to the Figshare platform once the article is published.

**Comment 3 – Journal requirements:**

Thank you for stating the following financial disclosure: Funding was provided by the Natural Sciences and Engineering Research Council of Canada (NSERC) through a Collaborative Research and Development grant awarded to C. Messier (RDCPJ: 498998-16) with the joint support of Resolute Forest Products, and a Discovery grant awarded to E.Filotas (RGPIN: 2018-06156).

Please state what role the funders took in the study. If the funders had no role, please state: ""The funders had no role in study design, data collection and analysis, decision to publish, or preparation of the manuscript.""

If this statement is not correct you must amend it as needed.

Please include this amended Role of Funder statement in your cover letter; we will change the online submission form on your behalf.

**Response:**

Thank you for notifying us of this necessity. We state that the funders had no role in study design, data collection and analysis, decision to publish, or preparation of the manuscript.

**Comment 4 – Journal requirements:**

In the online submission form, you indicated that your data will be submitted to a repository upon acceptance. We strongly recommend all authors deposit their data before acceptance, as the process can be lengthy and hold up publication timelines. Please note that, though access restrictions are acceptable now, your entire minimal dataset will need to be made freely accessible if your manuscript is accepted for publication. This policy applies to all data except where public deposition would breach compliance with the protocol approved by your research ethics board. If you are unable to adhere to our open data policy, please kindly revise your statement to explain your reasoning and we will seek the editor's input on an exemption.

**Response:**

We apologize for the confusion; our data was already uploaded at the moment of the submission and is still available at the following link: <https://figshare.com/s/8a7e11afb70e3340a4f4> . The key difference is that the data repository is currently private (can only be found and accessed through the link). It will become public (can be accessed without the link through different search engines) once the article is published. As long as the link was provided to the reviewers and will be indicated in the manuscript, then the data can already be accessed openly through it.

**Comment 5 – Journal requirements:**

We note that Figure 1 in your submission contain [map/satellite] images which may be copyrighted. All PLOS content is published under the Creative Commons Attribution License (CC BY 4.0), which means that the manuscript, images, and Supporting Information files will be freely available online, and any third party is permitted to access, download, copy, distribute, and use these materials in any way, even commercially, with proper attribution. For these reasons, we cannot publish previously copyrighted maps or satellite images created using proprietary data, such as Google software (Google Maps, Street View, and Earth). For more information, see our copyright guidelines: http://journals.plos.org/plosone/s/licenses-and-copyright.

We require you to either (1) present written permission from the copyright holder to publish these figures specifically under the CC BY 4.0 license, or (2) remove the figures from your submission:

a. You may seek permission from the original copyright holder of Figure 1 to publish the content specifically under the CC BY 4.0 license.

We recommend that you contact the original copyright holder with the Content Permission Form (http://journals.plos.org/plosone/s/file?id=7c09/content-permission-form.pdf) and the following text:

“I request permission for the open-access journal PLOS ONE to publish XXX under the Creative Commons Attribution License (CCAL) CC BY 4.0 (http://creativecommons.org/licenses/by/4.0/). Please be aware that this license allows unrestricted use and distribution, even commercially, by third parties. Please reply and provide explicit written permission to publish XXX under a CC BY license and complete the attached form.”

Please upload the completed Content Permission Form or other proof of granted permissions as an ""Other"" file with your submission.

In the figure caption of the copyrighted figure, please include the following text: “Reprinted from [ref] under a CC BY license, with permission from [name of publisher], original copyright [original copyright year].”

b. If you are unable to obtain permission from the original copyright holder to publish these figures under the CC BY 4.0 license or if the copyright holder’s requirements are incompatible with the CC BY 4.0 license, please either i) remove the figure or ii) supply a replacement figure that complies with the CC BY 4.0 license. Please check copyright information on all replacement figures and update the figure caption with source information. If applicable, please specify in the figure caption text when a figure is similar but not identical to the original image and is therefore for illustrative purposes only.

The following resources for replacing copyrighted map figures may be helpful:

USGS National Map Viewer (public domain): http://viewer.nationalmap.gov/viewer/

The Gateway to Astronaut Photography of Earth (public domain): http://eol.jsc.nasa.gov/sseop/clickmap/

Maps at the CIA (public domain): https://www.cia.gov/library/publications/the-world-factbook/index.html and https://www.cia.gov/library/publications/cia-maps-publications/index.html

NASA Earth Observatory (public domain): http://earthobservatory.nasa.gov/

Landsat: http://landsat.visibleearth.nasa.gov/

USGS EROS (Earth Resources Observatory and Science (EROS) Center) (public domain): http://eros.usgs.gov/#

Natural Earth (public domain): http://www.naturalearthdata.com/

**Response:**

We thank the editor for their guidance on the matter. The satellite data we used on Fig 1 comes from the Sentinel satellite, and has been edited by the Ministry of Forests and Natural Resources of Quebec on the open data portal “Données Québec”, under a clear CC-BY 4.0 license (see <https://www.donneesquebec.ca/recherche/dataset/mosaique-satellites>). We have changed the caption of Fig 1 as followed to better reflect the attribution and the status of the copyright:

*Fig 1: Map showing the location and extent of our study area located in the Mauricie region of Quebec, Canada. Satellite data from the Sentinel satellite, and edited by the Ministry of Forests and Natural Resources of Quebec under a CC-BY 4.0 license (https://www.donneesquebec.ca/recherche/dataset/mosaique-satellites).*

**Comment 6 – Journal requirements:**

Please include captions for your Supporting Information files at the end of your manuscript, and update any in-text citations to match accordingly. Please see our Supporting Information guidelines for more information: http://journals.plos.org/plosone/s/supporting-information.

**Response:**

We thank the editor for their guidance. We have updated all references to appendices in our manuscript as “S1 Appendix A”, “S1 Appendix B”, and so on. We have also added a caption for S1 in the “Supporting information section” precising:

***S1****. appendices giving additional figures and details about the parameterization of LANDIS-II*

**Comment 7 – Journal requirements:**

Please review your reference list to ensure that it is complete and correct. If you have cited papers that have been retracted, please include the rationale for doing so in the manuscript text, or remove these references and replace them with relevant current references. Any changes to the reference list should be mentioned in the rebuttal letter that accompanies your revised manuscript. If you need to cite a retracted article, indicate the article’s retracted status in the References list and also include a citation and full reference for the retraction notice.

**Response:**

To our knowledge, none of the papers that we cite have been retracted. If such a thing happens before the final publication of the manuscript, we will delete the reference or notify the editor of our choice to keep the reference.

# 2. Reviewer 1

**Comment 8 – Reviewer 1:**

Dear authors,

Thank you for providing a thorough and insightful analysis of the TRIAD+ approach within forest management to enhance resilience to climate change and extreme disturbances. This study, utilizing LANDIS-II in the Mauricie region, is both timely and relevant as forest managers face increasing pressures to incorporate resilience alongside conservation and production goals.

Paper is well written, English is very good, I have no major remarks. I suggest the minor revision.

The language is appropriate with no flaws (however, not a native speaker). Please find some minor remarks bellow:

Title: /

Abstract: /

Keywords:

it would make sense to add: management approach, disturbances

**Response:**

We want to thank the reviewer for their precious and detailed insights concerning our manuscript. The edits suggested by their comments will certainly improve our article. We are very grateful for their time and for their thorough review.

As suggested, we have added the keywords “management approach” and “disturbances” to the submission.

**Comment 9 – Reviewer 1:**

Comments:

266 first mention of SBW abbreviation should be written in full and SBW in brackets. A latin name should be added where SBW is first mentioned

291 first mention of ANPP abbreviation

SC and CT in Table 1 can also be written in full name

417 is referencing to Table 1 correct?

Figure 4: slight thinner graph lines of different management scenarios and higher resolution of figure would be better

Figure 7 caption typo: Bart plots -> bar plots

**Response:**

We thank the reviewer for these suggestions. We have taken them all into account.

- The scientific name of the spruce budworm (*Choristoneura fumiferana*) is now indicated in the section 2.1 (Simulated area) where the spruce budworm is first mentioned in the manuscript (line 123). We have also added a mention of the equivalence of the acronym SBW with the term Spruce Budworm in section 2.3.D (Spruce Budworm) where the acronym is first used.
- We have added the signification of the acronym ANPP (Annual Net Primary Productivity) where the acronym is first used in section 2.3.E (Harvesting).
- We have added the full signification of SC and CT in table 1 to make the table more readable.
- The reference to Table 1 at line 417 was indeed a typo. We have changed the reference to Table 3, which is how it should have been written.
- We have increased the resolution of Figure 4. However, we would prefer to keep the current line width, as the same width is used for figure 5, and because line width is used to distinguish the curves from the BAU (wider) and TRIAD (thinner) scenarios in the legend.
- We have corrected the “Bart plot” typo in the caption of figure 7.

**Comment 10 – Reviewer 1:**

Discussion: you will find some supporting results (regarding effect of PlantFunct and climate effect) in a study by BLATTERT et al. 2024. (Managing European Alpine forests with close-to-nature forestry to improve climate change mitigation and multifunctionality)

**Response:**

We thank the reviewer for the suggestion of the reference. We have added Blattert et al. 2024 in the discussion, at the line discussing results of others studies that investigated the effect of planting species adapted to climate change.

# 3. Reviewer 2

**Comment 11 – Reviewer 2:**

This manuscript titled "Climate is stronger than you think: Exploring functional planting and TRIAD zoning for increased forest resilience to extreme disturbances" addresses a timely and relevant topic in forest management. The integration of resilience components into the TRIAD zoning approach and its evaluation using the LANDIS-II simulation model under various climate and disturbance scenarios provide a novel and interesting perspective. The writing style is clear, the introduction is detailed and engaging, and the methods, results, and discussion are well-structured and informative. The study's findings contribute valuable insights into adapting forest management strategies to the challenges posed by climate change and extreme disturbances. I have some concerns

Concerns Regarding Statistical Support for Results

One notable concern is the absence of statistical tests to support the observed differences in functional diversity and resilience indicators across scenarios. While the manuscript discusses differences between TRIAD+, classic TRIAD, and BAU scenarios, it is unclear whether these differences are statistically significant or within the range of variability expected under the model framework. Including statistical analyses suitable for simulated data would provide stronger evidence for the conclusions drawn and clarify the practical implications of the observed trends.

**Response:**

We thank Reviewer 2 for their enthusiasm regarding our manuscript and for questions they highlighted, which we believe will be mirrored by the future readers of the article.

We believe that statistical tests are not needed in the case of an experimental protocol based of model simulation like ours, as described by White et al. (2014). Indeed, because the scenarios are known to have distinct factors a priori, simply increasing the number of replicates will end up making any difference between scenarios “statistically significant”. As such, we adopted a descriptive analysis of the overall temporal dynamics and used the variability between replicates as an indication of potential uncertainty in our conclusions, rather than the basis of any statistical test. This approach is similar to our previous study (see Hardy et al. 2023).

However, we believe that this question highlighted by Reviewer 2 will be pondered by other readers. As such, we have added the following sentences to the manuscript, in section 2.4 (Data analysis):

*In the end, we simply compared the temporal trends and average values for each measure (BL, F̅D̅S, BFG, R, NC and RR) between the scenarios to infer the effects of our different factors. In addition, we use the variability observed between replicates as an indication of uncertainty steaming from the stochastic processes inside our model. We did not use any statistical tests, as the scenario were known to have distinct factors a priori, meaning that any increase in the number of replicates would end up making any difference between scenarios statistically significant [104].*

With reference 104 being White et al. (2014).

**Comment 12 – Reviewer 2:**

Inclusion of Additional Metrics for Forest Resilience

The manuscript evaluates forest resilience using three metrics: resistance, net change, and recovery time of mature biomass. These metrics are appropriate but could be complemented by including functional redundancy, an index derived from functional diversity, to provide a more comprehensive assessment of resilience. Functional redundancy reflects the ability of ecosystems to maintain functions despite species loss and could enhance the interpretation of how functional planting influences forest stability under disturbance. I encourage the authors to explore the potential for including this index in their analysis.

**Response:**

We thank Reviewer 2 for their suggestion, and agree that Functional Redundancy represents an interesting resilience indicator. However, computing functional redundancy would require an important work of re-analyzing our large dataset of results, which will most probably go beyond the delay allocated by the editor for re-submission. We also believe that our current study is already strong with the 6 different variables already discussed, and we do not think that adding Functional Redundancy would lead to any major different results and interpretation. Adding another variable will also make the paper overly long and complex.

We will however consider functional redundancy as a crucial variable in our next research.

**Comment 13 – Reviewer 3:**

Practical Implications of Functional Planting

The results indicate that functional planting provides only modest increases in forest resilience under the simulated scenarios. Given the limited impact observed, the authors should address the practical feasibility of implementing such management strategies, considering the effort, cost, and potential trade-offs involved. Is the modest increase in resilience sufficient to justify the additional efforts required for functional planting? A discussion on this point, perhaps in comparison to alternative or complementary management strategies, would strengthen the manuscript’s practical relevance.

Overall, this study is a significant contribution to forest management literature, and the methodological approach is commendable. Addressing the above concerns, particularly the inclusion of statistical tests and a deeper discussion on practical feasibility, will enhance the manuscript's scientific rigor and applicability.

**Response:**

We thank Reviewer 2 for this insight. We agree that our study questions both the feasibility and the efficacy of functional enrichment planting to increase forest resilience to future disturbances. However, we want to remain cautious as to not dismissing it completely in our conclusion, based on the fact that our study also highlighted several important limitations of current Forest Landscape Models such as LANDIS-II to model such management prescription.

Our current discussion already highlights the challenges associated with the approach – along with comparing it to other planting approaches - in section 4.6 (Guidelines and considerations for future studies). We have added the following lines to address Reviewer 2’s concerns and to clarify our position regarding functional planting:

*As such, while the limitations of our study do not allow us to dismiss functional planting as an effective or practical strategy to enhance forest resilience (see previous section), we believe that our results highlight caveats and difficulties that must be considered in future studies. We also believe that while the potentially beneficial effect of functional planting on forest resilience seems to be validated by our model, its importance and efficacy when compared to alternative strategies still remains to be explored, as our results show a relatively small effect.*

# References

Hardy, C., Messier, C., Boulanger, Y., Cyr, D., Filotas, É., 2023. Land sparing and sharing patterns in forestry: exploring even-aged and uneven-aged management at the landscape scale. Landsc Ecol 38, 2815–2838. <https://doi.org/10.1007/s10980-023-01742-7>

White, J.W., Rassweiler, A., Samhouri, J.F., Stier, A.C., White, C., 2014. Ecologists should not use statistical significance tests to interpret simulation model results. Oikos 123, 385–388. https://doi.org/10.1111/j.1600-0706.2013.01073.x
